# Long-term metal fume exposure assessment of workers in a shipbuilding factory

**DOI:** 10.1038/s41598-021-04761-z

**Published:** 2022-01-17

**Authors:** Ying-Fang Wang, Yu-Chieh Kuo, Lin-Chi Wang

**Affiliations:** 1 Department of Occupational Safety and Health, Chung Shan Medical University, 110 Sec. 1 Jianguo Road, Taichung City, 40201 Taiwan; 2 Department of Occupational Medicine, Chung Shan Medical University Hospital, 110 Sec. 1 Jianguo Road, Taichung City, 40201 Taiwan; 3 Department of Environmental and Occupational Health, College of Medicine, National Cheng Kung University, 138 Sheng-Li Rd, Tainan City, 70403 Taiwan; 4 Department of Environmental Engineering, Chung Yuan Christian University, 200 Chung Pei Road, Chung Li District, Taoyuan City, 32023 Taiwan; 5 Center for Environmental Risk Management, Chung Yuan Christian University, 200 Chung Pei Road, Chung Li District, Taoyuan City, 32023 Taiwan

**Keywords:** Environmental monitoring, Health occupations

## Abstract

This study aims to assess the metal fume exposure of welders and to determine exposure rates for similar exposure groups in a shipyard through the use of Near-field/Far-field (NF/FF) mathematical model and Bayesian decision analysis (BDA) technique. Emission rates of various metal fumes (i.e., total chromium (Cr), iron (Fe), lead (Pb), manganese (Mn), and nickel (Ni)) were experimentally determined for the gas metal arc welding and flux cored arc welding processes, which are commonly used in shipyards. Then the NF/FF field model which used the emission rates were further validated by welding simulation experiment, and together with long-term operation condition data obtained from the investigated shipyard, the predicted long-term exposure concentrations of workers was established and used as the prior distribution in the BDA. Along with the field monitoring metal fume concentrations which served as the likelihood distribution, the posterior decision distributions in the BDA were determined and used to assess workers’ long-term metal exposures. Results show that the predicted exposure concentrations (C_p_) and the field worker’s exposure concentrations (C_m_) were statistically correlated, and the high R^2^ (= 0.81–0.94) indicates that the proposed surrogate predicting method by the NF and FF model was adequate for predicting metal fume concentrations. The consistency in both prior and likelihood distributions suggests the resultant posterior would be more feasible to assess workers’ long-term exposures. Welders’ Fe, Mn and Pb exposures were found to exceed their corresponding action levels with a high probability (= 54%), indicating preventive measures should be taken immediately. The proposed approach provides a universal solution for conducting exposure assessment with usual limited number of personal exposure data.

## Introduction

The welding process is one which is frequently encountered in the shipbuilding industry. Welding operations produce gaseous and aerosol by-products containing a complex array of metals (e.g., iron, manganese, chromium, and nickel, etc.), metal oxides, and other chemical species volatilized from the welding rod and the flux material incorporated within it^1^. Recent studies have shown that inhalation of welding fumes can excessively cause respiratory damage, such as bronchitis, asthma^2^, lung function change^3^, increased lung cancer risk^4,5^, neurotoxicity^6^ and many other diseases. In particular, exposures to specific metals contained in welding fume is of concern in the industrial hygiene field. For example, iron exposures can lead to siderosis; zinc, copper, and magnesium exposures can cause metal fume fever; and chromium VI can not only cause severe irritation to the upper respiratory tract, but also could be carcinogenic to human beings^7^.

The contents of metal and pollutants in the emitted particles and their concentrations in welding fume during the welding process are affected by the involved welding procedures, the filler and the base material of the welding rods, and the presence of coatings^8,9^. Therefore, investigating the emission rates of the abovementioned pollutants under specific welding conditions is important to assess workers' exposures. Considering that chronic health effects were known to be associated with welding fume exposures, conducting long-term exposure assessments has become an important issue to ensure that shipyard welders can work in a healthy environment.

However, it should be noted that obtaining complete long-term exposure data directly from field sampling is quite difficult in the real world because of constraints such as the cost, workers' unwillingness, and interference to work practices, etc. Some researchers have suggested the use of models for predicting exposure concentrations to solve the above problems, such as Well-Mixed Room model, Two-Zone model, Diffusion models, Computational Fluid Dynamics models (CFD)^10^. Based on laboratory tests, researchers have found that the exposure concentrations predicted by the near field (NF) and far field (FF) model correlate well with the corresponding measured values, because NF contains the contaminant emission source and encompasses the worker’s breathing zone whose exposure is to be estimated and FF is expressed as the rest of the room^11^. However, the suitability of the aforementioned model has not been validated in occupational environment.

To date the Bayesian decision analysis (BDA) technique has been adopted by many researchers in determining exposure profiles based on a small amount of sampling data^12^ to conduct the long-term exposure. BDA provides a transparent method for incorporating the relative certainty of the information or data used to produce a judgment probability chart^13,14^. The use of BDA requires knowledge of both prior and likelihood exposure distributions for the targeted similar exposure group (SEG). Subsequently, the posterior exposure distribution can be obtained to describe the exposure profile of the target SEG^13^. In theory, the limited measured concentrations can be used to describe the likelihood exposure distribution in BDA. Many methodologies have been used by industrial hygienists to create exposure data for determining the prior exposure distribution, such as the expert system^12,15–17^, numerical model^17–19^, and surrogate exposure method^13,17,20^. It should be noted that the use of the expert system might lead to inaccurate estimations of the posterior distribution due to inherent large variations among experts^12,15–17^. If the numerical model is adopted, it is applicable only when environmental and workforce conditions are comparable to the boundary conditions of the involved numerical method^21^. As for the surrogate method, it requires the validation of the effectiveness of the surrogate in predicting exposures of interest.

**Figure 1:**
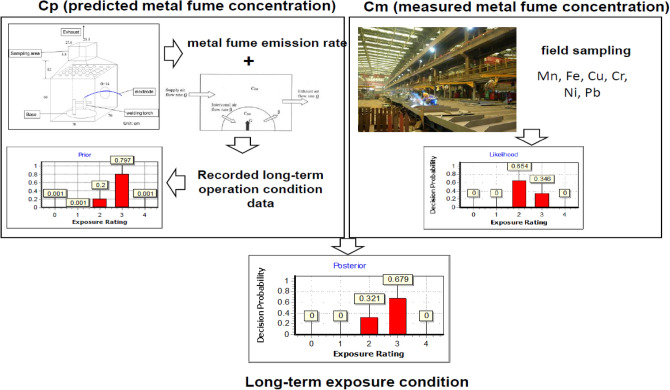
Graphic abstract of the research structure of this study.

The aim of the present study was to develop an effective and practical approach for conducting long-term exposure assessment for workers who exposed to welding fumes. First, the emission rates of various emitted metals in welding fumes under various welding operating conditions were determined. Secondly, the resultant emission rates were applied to the NF/FF field model for predicting exposure concentrations of various metals. The model results were further validated by comparing the predicted values with metal fume sampled and measured under the same welding operating conditions with NF/FF field model. After validation, workers' long-term predicted exposure concentrations were established using the NF/FF model and the recorded long-term operation condition data in the shipbuilding factory. Finally, the predicted long-term exposure concentrations and field monitoring metal fume concentrations of the shipbuilding factory served as the prior and likelihood distribution in the BDA, respectively. The resultant posterior distributions were used to characterize workers' long-term exposures to offer the basis for initiating control strategies for the shipbuilding factory to reduce metal fume exposures of welders. To better understand the research structure, a graphic abstract of this study is depicted in Fig. 1.

## Materials and methods

### Chamber experiment for determining metal fume emission rates

#### Chamber

The construction of the fume generation and collection apparatus used in the present study was according to the Japanese Industrial Standards Z 3930 (Method of measuring total amount of weld fumes generated by covered electrode). The fume collection chamber (Fig. S1) was 70 cm length × 70 cm wide × 60 cm height in magnitude, which consists of a ground steel workbench and a stainless-steel enclosure. Inside the chamber, arc welding is done with a welding robot which consists of a manipulator, a controller, and a power supply (ARCMAN-RON, KOBE STEEL, LTD., Japan). A base metal, 27 cm length × 27 cm wide × 1.2 cm height in magnitude, was placed at the center of the workbench rotating at a constant speed for each welding condition by a motor located outside of the chamber. Simultaneously, a wire alloy is fed at a constant speed to serve as the filler material for the arc welding process.

#### Selected welding operation conditions and sampling methods

Two welding processes which are most often used in the shipbuilding factory were selected, including (1) gas metal arc welding (GMAW) with KM56 (AWS ER 70S-6) and KM58 (AWS ER70S-G), and (2) flux cored arc welding (FCAW) with KFX71T (AWS E71T-1) and KFX70T (AWS E70T-1), respectively. According to the field observation, the arcing time, welding speed, and shield gas flow rate were set at 30 s, 28 cm/min, and 20 L/min, respectively. The wire (1.2 mm in diameter) extension and the torch angle were kept at 15 mm and 0°, respectively. Current intensities (Amperes) were set at 120, 220, and 300 Amperes, respectively.

The IOM personal inhalable aerosol samplers (SKC Inc., Eighty-four, PA, USA) were used to sample the metal fume. The location for collecting the emitted metal fume was at the breathing zone of the welder, which was set at a point 70 cm above the base metal. The sampling time for each testing condition was set at 300 s, including 30 s for arcing time, and the subsequent 240 s for the arc off period. Three repeated samplings were conducted for each selected welding processes. All collected samples were analyzed for selected metals by Inductively Coupled Plasma Atomic Emission Spectroscopy (ICP-AES) analysis following NIOSH Method 7300. The metals selected for analysis, including total chromium (Cr), iron (Fe), manganese (Mn), nickel (Ni), and lead (Pb), were based on the pre-tested samples obtained from the selected shipyard factory. The MDL for Cr, Fe, Mn, Ni, and Pb was 0.003 mg/sample, 0.004 mg/sample, 0.007 mg/sample, 0.009 mg/sample, and 0.01 mg/sample, respectively.

#### Estimated metal fume emission rate

In order to estimate the emission rate from welding processes, a mass balance equation has been proposed as the following^22^:

1 $$\frac{{\mathrm{dC}}_{\mathrm{in}}}{\mathrm{dt}}=\mathrm{p}\cdot \mathrm{AER}\cdot {\mathrm{C}}_{\mathrm{out}}+\frac{{\mathrm{Q}}_{\mathrm{s}}}{\mathrm{V}}+\left(\mathrm{AER}+\mathrm{k}\right)\cdot {\mathrm{C}}_{\mathrm{in}}$$

where ${\mathrm{C}}_{\mathrm{in}}$, ${\mathrm{C}}_{\mathrm{out}}$: the indoor and outdoor particle concentration; $\mathrm{p}$: the penetration efficiency; AER: air exchange rate; $\mathrm{k}$: the deposition rate; ${\mathrm{Q}}_{\mathrm{s}}$: the indoor particle generation rate; $\mathrm{t}$: time; $\mathrm{V}$: the efficient volume of the plenum. However, a simplified equation was adopted for estimating the emission rate as followings^23^:

2 $$\mathrm{ER}=\mathrm{V}\left[\frac{{\mathrm{C}}_{\mathrm{in}}-{\mathrm{C}}_{\mathrm{in},0}}{\Delta \mathrm{t}}+\left(\overline{\mathrm{AER }+\mathrm{k}}\right)\cdot \overline{{\mathrm{C} }_{\mathrm{in}}}-\mathrm{AER}\cdot {\mathrm{C}}_{\mathrm{in},0}\right]$$

where $\mathrm{ER}$: the average emission rate; ${\mathrm{C}}_{\mathrm{in}}, {\mathrm{C}}_{\mathrm{in},0}$: the peak and initial indoor particle concentrations; $\overline{\mathrm{AER }+\mathrm{k}}$: the average removal rate; $\Delta \mathrm{t}$: time difference between initial and peak concentration.

Because ${\mathrm{C}}_{\mathrm{in},0}$ is negligible with respect to ${\mathrm{C}}_{\mathrm{in}}$, Eq. (2) was simplified to Eq. (3):

3 $$\mathrm{ER}=\mathrm{V}\left[\frac{{\mathrm{C}}_{\mathrm{in}}}{\Delta \mathrm{t}}+\left(\overline{\mathrm{AER }+\mathrm{k}}\right)\cdot \overline{{\mathrm{C} }_{\mathrm{in}}}\right]$$

### Predicting and validating concentrations of metal fume during welding process

#### The Near-field and far-field model

In this study, the obtained ER was applied to the NF and FF model for predicting metal fume exposure concentrations (C_NF-p_ and C_FF-P_) according to Eqs. (4) to (7) (Nicas et al. 2006):

4 $${\mathrm{C}}_{\mathrm{N},\mathrm{t}}=\frac{\mathrm{ER}}{\mathrm{Q}}+\frac{\mathrm{ER}}{\upbeta }+\mathrm{ER}\left(\frac{\mathrm{\beta Q}+{\uplambda }_{2}{\mathrm{V}}_{\mathrm{N}}\left(\upbeta +\mathrm{Q}\right)}{\mathrm{\beta Q}{\mathrm{V}}_{\mathrm{N}}\left({\uplambda }_{1}-{\uplambda }_{2}\right)}\right){\mathrm{e}}^{{\uplambda }_{1}\mathrm{t}}-\mathrm{ER}\left(\frac{\mathrm{\beta Q}+{\uplambda }_{1}{\mathrm{V}}_{\mathrm{N}}\left(\upbeta +\mathrm{Q}\right)}{\mathrm{\beta Q}{\mathrm{V}}_{\mathrm{N}}\left({\uplambda }_{1}-{\uplambda }_{2}\right)}\right){\mathrm{e}}^{{\uplambda }_{2}\mathrm{t}}$$

5 $${\mathrm{C}}_{\mathrm{F},\mathrm{t}}=\frac{\mathrm{ER}}{\mathrm{Q}}+\mathrm{ER}\left(\frac{{\uplambda }_{1}{\mathrm{V}}_{\mathrm{N}}+\upbeta }{\upbeta }\right)\left(\frac{\mathrm{\beta Q}+{\uplambda }_{2}{\mathrm{V}}_{\mathrm{N}}\left(\upbeta +\mathrm{Q}\right)}{\mathrm{\beta Q}{\mathrm{V}}_{\mathrm{N}}\left({\uplambda }_{1}-{\uplambda }_{2}\right)}\right){\mathrm{e}}^{{\uplambda }_{1}\mathrm{t}}-\mathrm{ER}\left(\frac{{\uplambda }_{2}{\mathrm{V}}_{\mathrm{N}}+\upbeta }{\upbeta }\right)\left(\frac{\mathrm{\beta Q}+{\uplambda }_{1}{\mathrm{V}}_{\mathrm{N}}\left(\upbeta +\mathrm{Q}\right)}{\mathrm{\beta Q}{\mathrm{V}}_{\mathrm{N}}\left({\uplambda }_{1}-{\uplambda }_{2}\right)}\right){\mathrm{e}}^{{\uplambda }_{2}\mathrm{t}}$$

6 $${\uplambda }_{1}=0.5\left[-\left(\frac{\upbeta {\mathrm{V}}_{\mathrm{F}}+{\mathrm{V}}_{\mathrm{N}}\left(\upbeta +\mathrm{Q}\right)}{{\mathrm{V}}_{\mathrm{N}}{\mathrm{V}}_{\mathrm{F}}}\right)+\sqrt{{\left(\frac{\upbeta {\mathrm{V}}_{\mathrm{F}}+{\mathrm{V}}_{\mathrm{N}}\left(\upbeta +\mathrm{Q}\right)}{{\mathrm{V}}_{\mathrm{N}}{\mathrm{V}}_{\mathrm{F}}}\right)}^{2}-4\left(\frac{\mathrm{\beta Q}}{{\mathrm{V}}_{\mathrm{N}}{\mathrm{V}}_{\mathrm{F}}}\right)}\right]$$

7 $${\uplambda }_{2}=0.5\left[-\left(\frac{\upbeta {\mathrm{V}}_{\mathrm{F}}+{\mathrm{V}}_{\mathrm{N}}\left(\upbeta +\mathrm{Q}\right)}{{\mathrm{V}}_{\mathrm{N}}{\mathrm{V}}_{\mathrm{F}}}\right)-\sqrt{{\left(\frac{\upbeta {\mathrm{V}}_{\mathrm{F}}+{\mathrm{V}}_{\mathrm{N}}\left(\upbeta +\mathrm{Q}\right)}{{\mathrm{V}}_{\mathrm{N}}{\mathrm{V}}_{\mathrm{F}}}\right)}^{2}-4\left(\frac{\mathrm{\beta Q}}{{\mathrm{V}}_{\mathrm{N}}{\mathrm{V}}_{\mathrm{F}}}\right)}\right]$$

where ${\mathrm{C}}_{\mathrm{N}.\mathrm{t}}$ and ${\mathrm{C}}_{\mathrm{F},\mathrm{t}}$: the time-varying concentrations in the near field and in the far field, respectively; ER: emission rate; Q: room supply air rate; β: air flow rate between the near and far fields; ${\mathrm{V}}_{\mathrm{N}}$ and ${\mathrm{V}}_{\mathrm{F}}$: the near-field and far-field volumes; ${\uplambda }_{1}$ and ${\uplambda }_{2}$: the model parameter corresponding to air turnover rates.

#### Determining metal fume concentrations in welding simulation experiment

The schematic of the air samplings at NF and FF regions during welding processes is shown in Fig. S2, which was intended to simulate metal fume exposure concentrations of workers. To validate the predicted concentration by NF and FF model, the concentrations (C_m_) at NF (the hemisphere space with a radius 0.6 m away from the emission source) and FF regions (the space between the two hemispheres with a radius 0.6 m and 1.5 m away from the emission source) were measured for each selected welding processes for three repeated times^24^.

Three IOM personal inhalable aerosol samplers (SKC Inc., Eighty-four, PA, USA) were used to sample simultaneously at A1, B1 and C1 for NF region concentrations, while another three IOM personal inhalable aerosol samplers were deployed at A2, B2 and C2 for FF region concentrations. The welding time was 30 min. The sampling flow rate was specified at 2.0 L/min and the sampling time was for 30 min. All collected samples were analyzed for the selected metals by a Inductively Coupled Plasma Mass Spectrometry (ICP-MS) following NIOSH Method 7303. The LOQ for each metal element was 0.003 μg.

#### Statistical analysis

Simple liner regression analyses were used to examine the relationship between the predicted concentration (C_p_) and measured concentration (C_m_) following Eqs. (8) and (9):

8 $${\mathrm{C}}_{\mathrm{NF}-\mathrm{m}}={\mathrm{\alpha }}_{1}{\mathrm{C}}_{\mathrm{NF}-\mathrm{p}}+{\upbeta }_{1}$$

9 $${\mathrm{C}}_{\mathrm{FF}-\mathrm{m}}={\mathrm{\alpha }}_{2}{\mathrm{C}}_{\mathrm{FF}-\mathrm{p}}+{\upbeta }_{2}$$

If good correlations could be found between C_p_ and C_m_, the above regression equations could be used to further predict the long-term metal fume exposure concentrations for welding workers based on the records of the welding type and flow rate adopted in the working area.

Through field observation for 30 days, only 25 working days were in accordance with the welding type and wire selected in this study. The parameters based on the records of these 25 working days were used in NF-FF model via the use of the Monte Carlo simulation to predict the long-term exposure concentrations of shipyard workers in NF and FF regions. We used @risk software (Palisade Corporation, Version 7.0) to perform 100,000 times of simulation estimation.

In this study, the Monte Carlo simulation distribution of the exposure concentrations is used as the prior distribution, and the measured data in the shipyard factory is used as the likelihood distribution to obtain long-term exposure posterior distribution.

### Field monitoring on metal fume exposure concentrations of workers in a shipyard factory

Personal samples collected from 18 randomly selected welders were assumed to be representative to field NF region exposure concentrations. In addition, static samples (~ 1.5 m above the ground level at the location nearest to the 18 randomly selected worker) were assumed to be representative of field FF region exposure concentrations. On the sampling day, among these 18 selected welders, 12 workers performed FCAW using 220 A current intensities and KFX71T as the welding wire, and the other six workers performed GMAW using 220 A current intensities and KM56 as the welding wire. The wind speed was about 0.54 m/s (range 0.03–3.0 m/s) in the NF area and 0.47 m/s (range 0.07–2.2 m/s) in the FF area. To reduce the uncertainty, the welding operation conditions in field samplings were within those of the welding simulation experiment. Furthermore, through workplace observation, we can confirm that only welding operations are performed in shipbuilding sites during field sampling to reduce the possible influence of metal fume from other activities.

The IOM personal inhalable aerosol sampler (SKC Inc., Eighty-four, PA, USA) was used to measure the exposure concentration of metal fume with a sampling flow rate specified at 2.0 L/min and the sampling time for ~ 8 h. Airflow rates between the NF and FF were determined by an anemometer (TSI Inc., Model 9535, St. Paul, MN, USA). All field samples from the shipyard factory were analyzed for the selected metals (Cr, Fe, Mn, Ni and Pb) by a Inductively Coupled Plasma Mass Spectrometry (ICP-MS) following NIOSH Method 7303. The LOQ for each metal element was 0.003 μg.

This study was approved by the Human Ethics Committee of the Chung Shan Medical University Hospital with respect to scientific content. We had adherence to relevant ethical guidelines/regulations. A written consent document containing the required information was provided to each subject and the informed consent was obtained from each subject involved in the study.

### Using BDA for long-term exposure assessment

In the present study, the predicted long-term metal fume exposure concentrations (C_p_) and the field workers' exposure concentrations (C_m_) were used for establishing the prior and likelihood exposure distributions in BDA, respectively and the resulting posterior exposure distributions were used to assess the long term exposures of workers. The software of the IH Data Analyst V1.27 (Exposure Assessment Solutions, Inc., Morgantown, West Virginia, USA) was used for conducting BDA. The 8-h time-weighted-average threshold limit values (TLV-TWA) of Cr, Fe, Mn, Ni, and Pb, which were 0.5 mg/m^3^, 10 mg/m^3^, 0.1 mg/m^3^ (inhalable particulate matter), 1 mg/m^3^, and 0.05 mg/m^3^ were chosen as their occupational exposure limit (OEL). The exposure ratings were classified into five categories: ER0 $\le$ 0.005 occupational exposure limit (OEL), 0.005OEL $<$ ER1 $\le$ 0.05OEL, 0.05OEL $<$ ER2 $\le$ 0.25OEL, 0.25OEL $<$ ER3 $\le$ 0.5OEL, and ER4 $>$ 0.5OEL, respectively. We regard one-half of the selected OEL as the action level. This means that if the exposure reaches the action level, the employer has to take action and implement exposure control measures to reduce the exposure potential. The recommended controls corresponding to each category are no action for ER0, general hazardous communication for ER1, chemical-specific hazardous communication for ER2, exposure surveillance/medical surveillance/work practices for ER3, and respirators/work practice controls for ER4, respectively.

## Results and discussion

### Emission rates of metal fume for the selected welding processes

The emission rates (ER) of the welding metal fume obtained from the chamber experiment are listed in Table 1. Fe and Mn exhibited the highest ERs (= 20.5–72.9 mg/min and 1.91–9.35 mg/min, respectively), and were much larger than those of Cr, Ni, and Pb (range = 0.02–0.11 mg/min). The above results can be explained by the relative compositions of the electrodes and were consistent with the findings reported in other study^25^. We also found that the ERs increased as the current intensity increased, which reflected the fact that a higher arc temperature results in a higher fume emission rate. However, the increase in ER is not linear with time due to the variations in time spent in different metal transfer modes. Some studies have reported the same results for various welding types, for instance, gas metal arc^26–28^. It is also known that metal transfer modes are intrinsically related to both the current intensity and voltage at the tip of the electrode. A previous study indicated that fume rates rise with voltage as one moves from short circuit (low voltage) to globular transfer (the ridge), then drops into the valley during a shift toward spray mode and finally rises again with the onset of streaming (high voltage) transfer^28^.

**Table 1:** The emission rates (ER) (mg/min) (CV) of welding metal fume for the selected welding processes.

Fume type	Current (A)	FCAW		GMAW	
		KFX71T	KFX70T	KM56	KM58
Cr	120	0.02(0.87)	0.03(0.88)	0.02(2.16)	0.03(1.11)
	220	0.04(1.96)	0.04(1.62)	0.03(1.24)	0.03(1.92)
	300	0.07(1.23)	0.09(3.01)	0.06(2.16)	0.09(1.34)
Fe	120	25.2(1.26)	27.9(0.92)	20.5(1.88)	24.7(2.46)
	220	31.8(1.85)	36.2(1.14)	29.4(2.85)	35.1(1.92)
	300	69.8(1.02)	72.9(1.26)	64.7(2.16)	67.8(1.92)
Mn	120	1.91(1.03)	3.91(1.25)	1.93(2.25)	3.06(1.48)
	220	3.39(2.62)	6.23(1.93)	2.65(2.81)	3.45(2.07)
	300	8.01(1.44)	9.35(1.94)	7.94(1.24)	8.78(1.05)
Ni	120	0.02(2.04)	0.03(0.82)	0.03(2.14)	0.03(3.41)
	220	0.05(3.98)	0.08(1.62)	0.04(2.15)	0.05(1.81)
	300	0.11(2.83)	0.11(2.01)	0.06(3.66)	0.09(1.86)
Pb	120	0.02(1.45)	0.03(0.99)	0.02(2.49)	0.02(2.58)
	220	0.04(2.48)	0.03(1.40)	0.03(2.46)	0.03(2.01)
	300	0.09(2.07)	0.10(1.49)	0.06(1.64)	0.08(1.02)

### Predicted exposure concentrations by the NF and FF model

In this study, the obtained ERs (Table 1) were applied to the NF and FF models for predicting metal fume exposure concentrations. The ER values were treated as the generation rate G in the adopted NF and FF models. The interflow term β between the NF and FF region is shown in Table S1, and the average β fell to 6.78–11.1 m^3^/min for the FCAW welding processes, and 5.11–9.08 m^3^/min for GMAW welding processes, respectively. As for other parameters used in NF and FF models, they were obtained according to the field measured data, and are listed in Table S2.

The predicted concentrations (C_p_) of the five metal fume elements (Cr, Fe, Mn, Ni, and Pb) obtained by NF and FF models for the selected welding processes are listed in Table S3. It showed that the predicted concentrations all increased as the applied current increased. Fe and Mn were the two elements with highest concentrations. The above results are consistent with the measured emission rates (Table 1). The mean predicted concentration of each metal in the NF was consistently higher than the corresponding value in FF. Spearman's correlation analyses (r = 0.97, p < 0.001) show significant correlations between the NF and FF predicted concentrations.

### Validation of the exposure concentrations obtained from NF and FF models

The measured NF and FF metal concentrations (C_m_) obtained from the welding simulations for two welding processes are shown in Table 2. Fe and Mn have their measured NF concentrations above the exposure limit adopted by ACGIH (TLV-TWA), 10 mg/m^3^ and 0.1 mg/m^3^ (inhalable particulate matter), respectively. As for the measured FF concentrations, Fe was generally above the TLV. These results revealed that exposure to metal fume concentrations for welding workers could be severe. Similarly, the measured metal concentrations all increased as the applied current increased, and Fe and Mn were the two elements with the highest concentrations. The above results are consistent with Table 1 and Table S3. Moreover, Spearman's correlation (r = 0.98, p < 0.001) also showed that there is a significant correlation between the NF and FF measured concentrations. By comparing the C_p_ with C_m_, it is apparent that most of the C_p_ are underestimated, with the exception of Mn. The underestimation maybe mainly comes from the two factors used in the models, the airflow rates, Q and β (Table S2), which were determined by anemometers. That is because the shipyard factories are usually naturally ventilated, resulting in a large range of wind speed variation on site. Therefore, using field-measured data to validate predicted concentrations by model is important.

**Table 2:** The measured NF and FF metal concentrations (C_m_) obtained from the welding simulations for two welding processes (mg/m^3^).

Fume type	Current (A)	FCAW				GMAW			
		KFX71T		KFX70T		KM56		KM58	
		NF	FF	NF	FF	NF	FF	NF	FF
Cr	120	0.007	0.002	0.011	0.004	0.003	0.001	0.004	0.002
	220	0.008	0.004	0.016	0.007	0.004	0.002	0.005	0.002
	300	0.011	0.005	0.018	0.009	0.006	0.003	0.006	0.003
Fe	120	22.9	7.15	20.6	8.21	16.1	5.95	20.5	7.69
	220	26.9	13.6	30.7	13.2	20.0	9.17	23.4	11.4
	300	37.9	17.8	34.5	17.1	29.1	15.6	28.7	15.8
Mn	120	0.184	0.057	0.261	0.104	0.241	0.089	0.308	0.116
	220	0.215	0.108	0.389	0.168	0.301	0.138	0.351	0.172
	300	0.303	0.142	0.438	0.217	0.438	0.234	0.431	0.238
Ni	120	0.005	0.001	0.011	0.004	0.003	0.001	0.004	0.002
	220	0.005	0.003	0.016	0.007	0.004	0.002	0.005	0.002
	300	0.008	0.004	0.018	0.009	0.006	0.003	0.006	0.003
Pb	120	0.005	0.001	0.008	0.003	0.003	0.001	0.004	0.002
	220	0.005	0.003	0.012	0.005	0.004	0.002	0.005	0.002
	300	0.008	0.004	0.014	0.007	0.006	0.003	0.006	0.003

All these predicted metal fume exposure concentrations served as a basis for establishing the long-term exposure data bank. Table S4 shows that simple linear regression analyses can reliably relate C_p_ and their corresponding C_m_. All resultant regression coefficients were positive and were statistically significant (p < 0.05). The high R^2^ (= 0.81–0.94) indicates that the proposed surrogate predicting method was adequate for predicting metal fume concentrations.

### Establishing long-term exposure concentrations of workers

After the relation of C_p_ and C_m_ was established and C_p_ was validated, the predicted long-term exposure concentrations of workers can be established using the recorded long-term operation condition data. Therefore, the field observation (30 day records from the investigated shipyard factory) and the developed NF and FF models were used to establish the predicted long-term exposure concentrations of workers in the shipyard factory. In other words, the parameters listed in Table S2 were obtained for the records of 25 working days which were in accordance with the welding type and wire selected in this study. Then these parameters were used in NF-FF model via the use of the Monte Carlo simulation to predict the long-term exposure concentrations of shipyard workers in NF and FF regions.

The geometric mean (GMs) and geometric standard deviations (GSDs) of the predicted long-term exposure concentrations of shipyard workers in NF and FF regions are listed in Table 3. Results show that the predicted Mn concentrations (0.236 mg/m^3^) for NF was higher than the OEL, 0.1 mg/m^3^, (inhalable particulate matter) promulgated by both the US and Taiwan OSHA. When using the NF-FF model to predict, β is one of the important parameters in the model, and the β value will determine the magnitude of the predicted concentration. When the NF-FF model was used to predict the concentration in NF region, it will be affected by the sampling position and β. The predicted value of FF was less affected by β, so the GSD of the concentrations in FF region was smaller.

**Table 3:** The GMs and GSDs (mg/m^3^) of the predicted long-term exposure concentrations of shipyard workers in NF and FF regions.

	NF (n = 25)		FF (n = 25)	
	GM	GSD	GM	GSD
Cr	0.008	1.32	0.005	1.09
Fe	6.13	1.35	1.22	1.08
Mn	0.236	1.16	0.098	1.06
Ni	0.009	1.92	0.003	1.11
Pb	0.003	1.41	0.002	1.08

### Assessing the long-term metal fume exposure profile for welding workers

The GMs and GSDs (mg/m^3^) of the field metal fume concentrations in the NF and FF regions of the welders in the investigated shipbuilding factory are shown in Table 4. As shown in Table 4, the GMs of Mn in NF and FF regions (1.38 and 0.196 mg/m^3^) were above the OEL, and the results were similar to those found in other studies conducted for shipyard welding workers (= 0.004–2.67 mg/m^3^)^29^. Then the predicted long-term exposure concentrations and this field monitoring metal fume concentrations can serve as the prior and likelihood distribution in the BDA, respectively, to obtain the posterior distributions for the long-term metal fume exposure of the welding workers.

**Table 4:** The GMs and GSDs (mg/m^3^) of the field metal fume concentrations in the NF and FF regions of the welders.

	NF (n = 18)		FF (n = 18)	
	GM	GSD	GM	GSD
Cr	0.064	2.68	0.013	2.44
Fe	5.31	1.93	1.06	2.58
Mn	1.38	1.97	0.196	2.16
Ni	0.011	2.34	0.001	2.17
Pb	0.008	2.28	0.005	2.56

**Figure 2:**
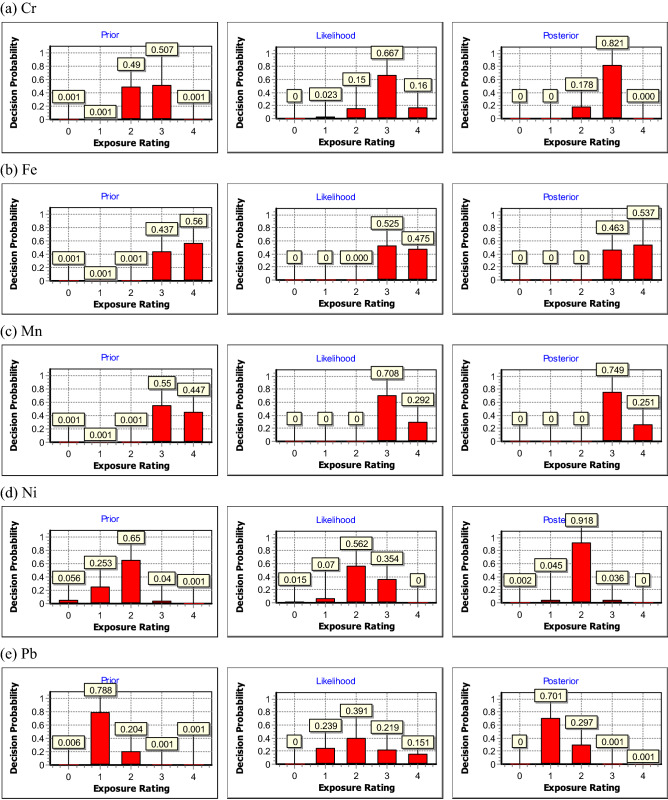
The resultant prior, likelihood, and posterior distributions for (**a**) Cr, (**b**) Fe, (**c**) Mn, (**d**) Ni, and (**e**) Pb fume concentrations in NF region, respectively.

**Figure 3:**
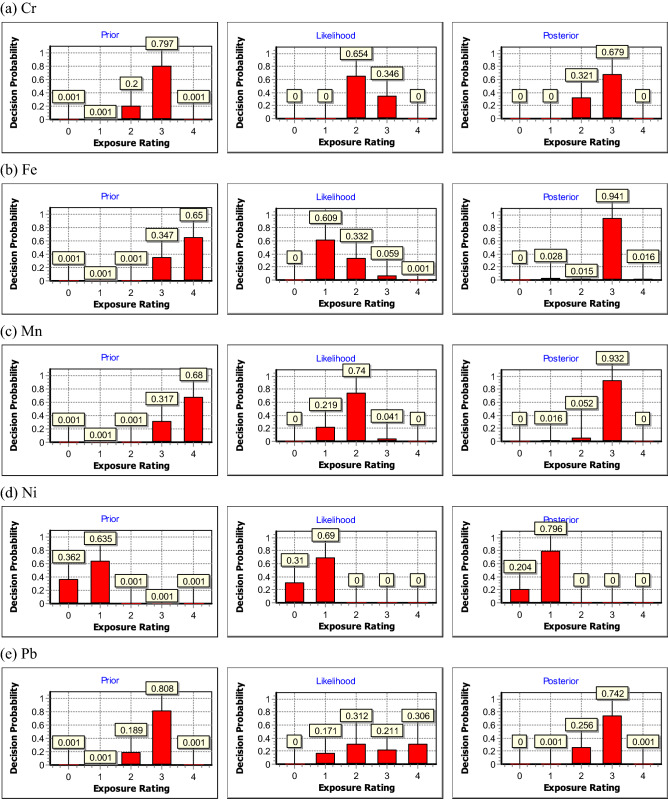
The resultant prior, likelihood, and posterior distributions for (**a**) Cr, (**b**) Fe, (**c**) Mn, (**d**) Ni, and (**e**) Pb fume concentrations in FF region, respectively.

Figures 2 and 3 show the prior, likelihood, and posterior distributions for Cr, Fe, Mn, Ni, and Pb concentrations in the NF and FF regions, respectively. As shown in Figs. 2 and 3, for prior and likelihood distributions, the dominant probabilities of Fe and Mn mainly fell to ER3 and ER4 in NF and FF regions, while those of Cr were at ER2 and ER3, and those of Ni were mostly at ER0, ER1 and ER2 in NF and FF regions. As for Pb, its dominant probabilities of prior distribution were distributed at ER1 and ER2 in NF region but ER2 and ER3 in FF region, while its likelihood distribution were evenly distributed. The above results shows that these metals have different prior distributions in the NF and FF regions. Therefore, using total metal concentration in the fume to directly assess welding workers' exposure profiles, which was commonly seen, could be inappropriate and might underestimate welding workers' exposures. Based on the results of this study, we suggested that it is more appropriate to use the fume concentration of each metal to assess welder's exposure. Furthermore, it should be noted that the dominant probabilities between the prior and likelihood distributions for some metals may be inconsistent, maybe because only limited field samples were measured in the shipbuilding factory. The consistency in both prior and likelihood distributions suggests the resultant posterior would be more feasible to assess workers’ long-term exposures^21^. Because the prior and likelihood distributions of Fe and Mn in the NF region shared the same trend, it is suggested that Fe and Mn concentrations be the primary indicator to assess welders’ exposures.

For the posterior distributions which were used to assess welding workers' long-term metal fume exposure profiles, the probabilities of Fe, Mn, and Pb in the NF region were at ER4 with values of 54%, 25%, 0.1%, while Fe and Pb in the FF region were at ER4 with values of 1.6%, and 0.1% respectively, indicating that the above metals should not be ignored since the exposure concentrations could be greater than the action level (0.5 OEL). The dominant probabilities of the posterior distribution in the NF region (Fig. 2) were at ER3 (= 82%) for Cr, ER4 (= 54%) for Fe, ER3 (= 75%) for Mn, ER2 (= 92%) for Ni and ER1 (= 70%) for Pb, while those in the FF region (Fig. 3) were all at ER3, except for Ni (ER1 = 80%). These results revealed that the welding workers were indeed exposed to excessive metal fume in both NF and FF regions. Therefore, appropriate control measures should be taken by the shipyard manufacturing industry, such as installing a mobile local exhaust ventilation system, and providing suitable personal respiratory protective equipment for welders.

## Conclusions

In this study, we found that the NF and FF models were suitable for predicting metal concentrations in welding fume. Using the month-round daily predicted concentrations and field monitoring concentrations in a shipyard manufacturing factory as the prior and likelihood distributions in the BDA, the resultant posterior distributions could be effectively applied to assess the long-term exposures of welders. We found that welders’ long-term Fe, Mn and Pb exposures were, probability, to exceed the action level. It is concluded that preventive measures should be taken for reducing welders’ exposures immediately. In addition, it is also found that Fe and Mn have the same trends of prior and likelihood distributions in different exposure regions. Therefore, it is suggested that both Fe and Mn concentrations could be used to replace the total fume concentrations to assess welders’ exposure. The proposed integrated approach can provide a universal solution for conducting exposure assessment with usual limited number of personal exposure data. However, it should be noted that the proposed integrated approach is better applicable when the operating conditions in the field are similar to those in the simulation experiments, and other activities that could result in similar pollutant emissions should be avoided in the field.

## Supplementary Information

Supplementary Information.
