# Supplementary material for: Long-term metal fume exposure assessment of workers in a shipbuilding factory
Source: Sci Rep. 2022 Jan 17;12:790. doi: 10.1038/s41598-021-04761-z (PMC8763891; doi:10.1038/s41598-021-04761-z)
Supplement: Supplementary file 1 — Supplementary Information. [file 41598_2021_4761_MOESM1_ESM.docx]

**Supplementary Materials for**

**Long-term metal fume exposure assessment of workers in a** **shipbuilding factory**

Ying-Fang Wang^1,2^, Yu-Chieh Kuo^3^, Lin-Chi Wang^4,5^*

*^1^ Department of Occupational Safety and Health, Chung Shan Medical University, 110 Sec. 1 Jianguo Road, Taichung City 40201, Taiwan.*

*^2^* *Department of Occupational Medicine, Chung Shan Medical University Hospital, 110 Sec. 1 Jianguo Road, Taichung City 40201, Taiwan.*

*^3^Department of Environmental and Occupational Health, College of Medicine, National Cheng Kung University, 138 Sheng-Li Rd, Tainan City 70403, Taiwan.*

*^4^ Department of Environmental Engineering, Chung Yuan Christian University, 200 Chung Pei Road, Chung Li District, Taoyuan City, 32023, Taiwan*

*^5^ Center for Environmental Risk Management, Chung Yuan Christian University, 200 Chung Pei Road, Chung Li District, Taoyuan City, 32023, Taiwan*

**^*^Corresponding author:** Tel: +886-3-265-4913; E-mail: [lcwang@cycu.edu.tw](mailto:lcwang@cycu.edu.tw)


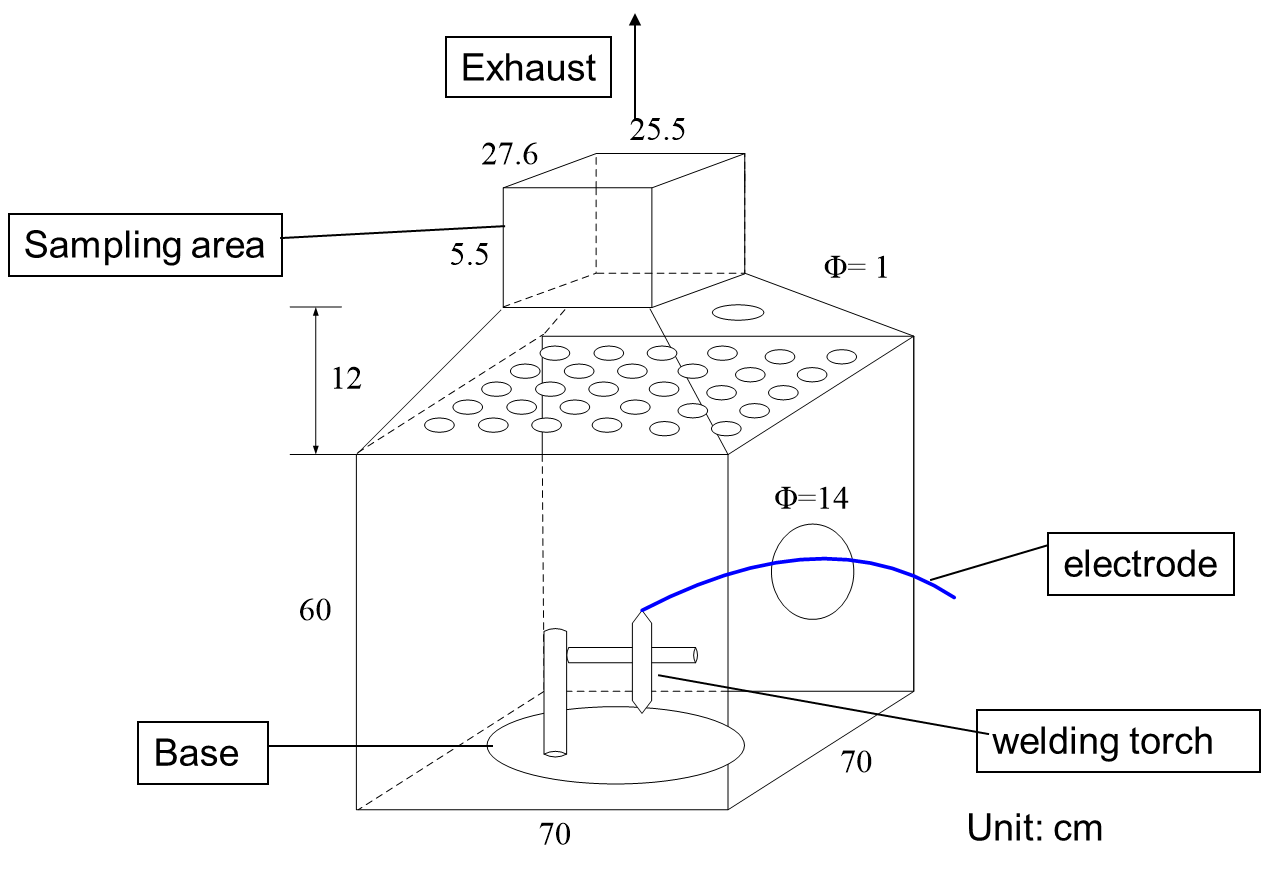


Fig. S1 The welding fume generation and collection chamber


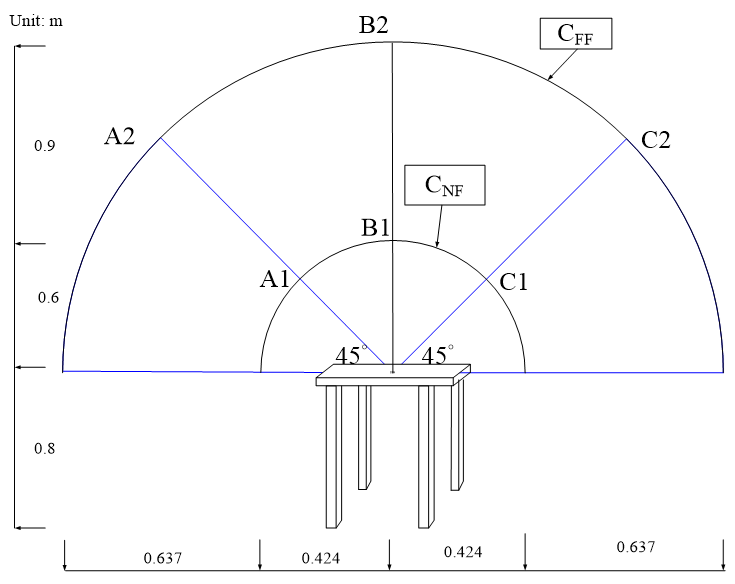


Fig. S2 Schematic of the air samplings at NF and FF regions in welding simulation experiment

Table S1 The air flow rate (β) (m^3^/min)(SD) between the near and far fields by field measurement from the selected welding processes.

| Current (A) |  | FCAW | | |  | GMAW | | |
| --- | --- | --- | --- | --- | --- | --- | --- | --- |
|  |  | KFX71T  (n=3) |  | KFX70T  (n=3) |  | KM56  (n=3) |  | KM58  (n=3) |
| 120 |  | 7.12(1.96) |  | 6.78(1.94) |  | 5.43(1.82) |  | 5.11(1.63) |
| 220 |  | 7.69(1.76) |  | 8.82(2.05) |  | 6.11(1.76) |  | 7.46(1.81) |
| 300 |  | 10.3(1.92) |  | 11.1(2.07) |  | 8.82(1.96) |  | 9.08(2.19) |

Table S2 Parameters used in NF-FF model

| Parameter | Symbol | Unit | Value | Reference |
| --- | --- | --- | --- | --- |
| Room Supply Flow Rate | Q | m^3^/min | 22.3 | Field measurement |
| Generation Rate | G | mg/min | Table 1 | Sampling |
| Near Field Volume | V_N_ | m^3^ | 0.45 | Field observation |
| Far Field Volume | V_F_ | m^3^ | 44.8 | Field observation |
| Flow Rate between NF and FF regions | β | m^3^/min | Table S1 | Field measurement |

Table S3 The predicted concentrations (C_p)_ obtained by NF-FF model from the selected welding processes (mg/m^3^). (n=3)

| Fume type |  | Current (A) |  |  | FCAW | | | | |  |  | | GMAW | | | | | |
| --- | --- | --- | --- | --- | --- | --- | --- | --- | --- | --- | --- | --- | --- | --- | --- | --- | --- | --- |
|  |  |  |  | KFX71T | | |  | KFX70T | | | |  | | KM56 | |  | KM58 | |
|  |  |  |  | NF | | FF |  | NF | FF | | |  | | NF | FF |  | NF | FF |
| Cr |  | 120 |  | 0.003 | | 0.001 |  | 0.005 | 0.001 | | |  | | 0.002 | 0.001 |  | 0.007 | 0.001 |
|  |  | 220 |  | 0.006 | | 0.002 |  | 0.006 | 0.002 | | |  |  | 0.002 | 0.001 |  | 0.005 | 0.001 |
|  |  | 300 |  | 0.009 | | 0.003 |  | 0.011 | 0.004 | | |  |  | 0.004 | 0.002 |  | 0.013 | 0.003 |
| Fe |  | 120 |  | 4.41 | | 0.98 |  | 5.07 | 1.09 | | |  | | 4.42 | 0.79 |  | 5.60 | 0.95 |
|  |  | 220 |  | 5.25 | | 1.24 |  | 5.39 | 1.42 | | |  |  | 5.78 | 1.14 |  | 5.91 | 1.37 |
|  |  | 300 |  | 9.34 | | 2.74 |  | 9.29 | 2.87 | | |  |  | 9.65 | 2.53 |  | 9.91 | 2.66 |
| Mn |  | 120 |  | 0.332 | | 0.074 |  | 0.709 | 0.152 | | |  | | 0.416 | 0.075 |  | 0.693 | 0.118 |
|  |  | 220 |  | 0.559 | | 0.132 |  | 0.929 | 0.244 | | |  |  | 0.522 | 0.103 |  | 0.582 | 0.135 |
|  |  | 300 |  | 1.07 | | 0.314 |  | 1.19 | 0.367 | | |  |  | 1.19 | 0.311 |  | 1.28 | 0.344 |
| Ni |  | 120 |  | 0.003 | | 0.001 |  | 0.005 | 0.001 | | |  | | 0.006 | 0.001 |  | 0.007 | 0.001 |
|  |  | 220 |  | 0.008 | | 0.002 |  | 0.012 | 0.003 | | |  |  | 0.008 | 0.002 |  | 0.008 | 0.002 |
|  |  | 300 |  | 0.013 | | 0.004 |  | 0.014 | 0.004 | | |  |  | 0.009 | 0.002 |  | 0.013 | 0.004 |
| Pb |  | 120 |  | 0.003 | | 0.001 |  | 0.005 | 0.001 | | |  | | 0.004 | 0.001 |  | 0.005 | 0.001 |
|  |  | 220 |  | 0.007 | | 0.002 |  | 0.004 | 0.001 | | |  |  | 0.006 | 0.001 |  | 0.005 | 0.001 |
|  |  | 300 |  | 0.012 | | 0.004 |  | 0.013 | 0.004 | | |  |  | 0.009 | 0.002 |  | 0.012 | 0.003 |

Table S4 The simple linear regression model of predicted concentrations (C_p_) and their corresponding measured concentrations (C_m_).

|  |  | NF | | |  | FF | | |
| --- | --- | --- | --- | --- | --- | --- | --- | --- |
|  |  | Predicted model |  | R^2^ |  | Predicted model |  | R^2^ |
| Cr |  | C_m_=1.29C_p_+0.001 |  | 0.85 |  | C_m_=2.36C_p_-0.0002 |  | 0.87 |
| Fe |  | C_m_=3.42C_p_+3.81 |  | 0.81 |  | C_m_=4.82C_p_+3.95 |  | 0.85 |
| Mn |  | C_m_=0.77C_p_+0.13 |  | 0.83 |  | C_m_=1.46C_p_+0.05 |  | 0.82 |
| Ni |  | C_m_=2.11C_p_-0.001 |  | 0.94 |  | C_m_=2.28C_p_-0.0001 |  | 0.94 |
| Pb |  | C_m_=1.06C_p_-0.001 |  | 0.82 |  | C_m_=1.56C_p_+0.0004 |  | 0.91 |
